# The Fate of the Plant Embryo's Suspensor: Balancing Life and Death

**DOI:** 10.1371/journal.pbio.1001656

**Published:** 2013-09-10

**Authors:** Charles Q. Choi

**Affiliations:** Freelance Science Writer, Flushing, New York, United States of America

The plant embryo is supported by a lifeline known as a suspensor that acts like an umbilical cord, connecting the embryo to surrounding tissues in the seed and ferrying in vital nutrients and growth factors. Although scientists have for a century known that the suspensor is a temporary organ, the molecular trigger for its demise was unknown. Now Meng-xiang Sun at Wuhan University in China and his colleagues have uncovered one key way this programmed cell death is controlled — a pair of proteins that antagonistically balance each other, with one spurring death and the other preventing it.

**Figure:**
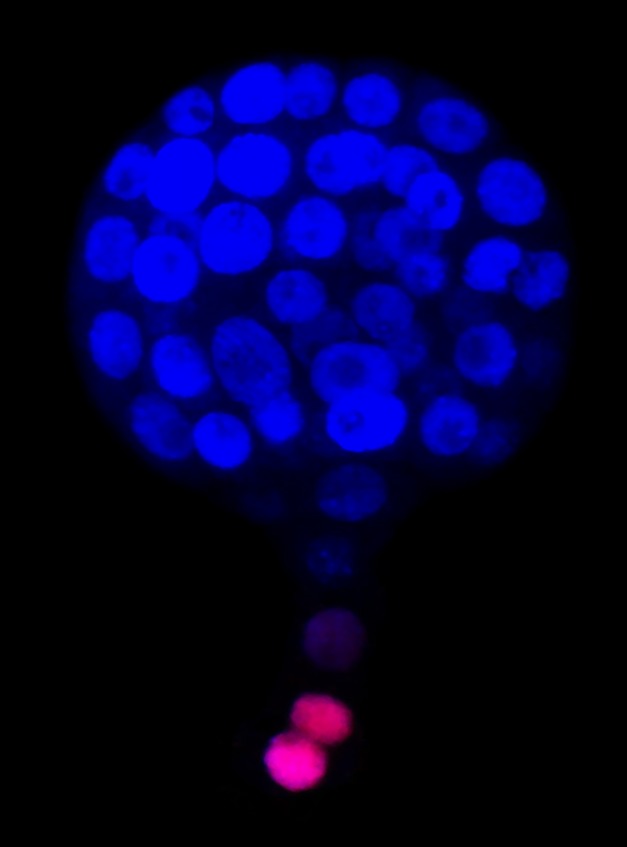
Plant embryos are supported by a temporary organ called a suspensor that connects embryos to surrounding nutrient-providing tissues. The suspensor dies early in embryonic development due to the actions of a protein pair that antagonistically balance each other, one being a pro-death cathepsin protease called NtCP14, the other an anti-death protease inhibitor called NtCYS.

After a plant egg cell is fertilized, it divides to become what is called a proembryo, which possesses two kinds of daughter cells with profoundly different fates. One, the smaller cell sitting at its tip, becomes the embryo, while the other, the larger cell resting at its base, develops into the suspensor that anchors the embryo to the parent plant. The suspensor, which occurs in a dazzling variety of shapes and sizes in the plant kingdom, is required only in the early stages of embryonic development — the disintegration of suspensor cells marks the earliest instance of programmed cell death in a plant's life.

To help solve the mystery behind the suspensor's downfall, researchers experimented with tobacco plants, whose suspensors are relatively easy to study, made up as they are of just simple columns of about four cells that develop through a well-known, precisely timed sequence of cell divisions. The suspensor begins to die once the embryo reaches the 32-celled stage, with death beginning at its base and progressing up to its tip, with all the contents of its cells slowly digested from within.

The scientists focused on a tobacco gene they named *NtCYS*. Genetic analysis revealed this gene is seen only in the basal cells that develop into suspensors — its activity is greatest during the eight- to 32-celled stage of the embryo, after which its levels decrease dramatically.

The researchers discovered the natural onset of suspensor degeneration coincided with a steady decrease in *NtCYS* activity. When they artificially silenced *NtCYS* activity with interfering RNA molecules early on in plant development, premature cell death occurred in the basal cell lineage, which in turn killed off embryos. By contrast, overexpressing *NtCYS* profoundly delayed cell death in the suspensor. All in all, these findings revealed *NtCYS* suppresses cell death.

*NtCYS* encodes a kind of protein known as a cystatin, which typically inhibit enzymes known as cysteine proteases. Genetic analysis revealed 20 genes encoding cysteine proteases that were active in the two-celled proembryo. Of those, NtCYS apparently interacted with six cathepsins, proteases linked with cell death; moreover, NtCYS was found in the same locations in the cell as one of those cathepsins named NtCP14, suggesting NtCYS targeted that enzyme.

The scientists discovered that overexpressing *NtCP14* triggered precocious cell death in the basal cell lineage, causing massive embryonic death, while silencing *NtCP14* delayed suspensor cell death. In other words, whereas NtCYS is anti-death, NtCP14 is pro-death.

It remains a puzzle as to what molecule or molecules naturally set off the rapid decrease of NtCYS activity that kills off suspensors. Since the ephemeral organ's programmed cell death starts at its base, the signal might come from cells at the micropyle, the opening through which the pollen tube penetrates into the ovule, the female part of the plant. Alternatively, the signal might come from the embryo itself, a possibility supported by the fact that suspensor programmed cell death always starts when the embryo reaches a very specific point in its life, its 32-celled stage.

In addition to analyzing what controls the activity of NtCYS and NtCP14, future research can also pinpoint what they control. The investigators discovered that increasing NtCP14 activity in the two-celled proembryo correspondingly increased the activity of molecules similar to caspases, enzymes linked with programmed cell death in animals.

**Zhao P, Zhou X-M, Zhang L-Y, Wang W, Ma L-G, et al. (2013) A Bipartite Molecular Module Controls Cell Death Activation in the Basal Cell Lineage of Plant Embryos. doi: 10.1371/journal.pbio.1001655**

